# Dynamic Virtual Fixture Generation Based on Intra-Operative 3D Image Feedback in Robot-Assisted Minimally Invasive Thoracic Surgery

**DOI:** 10.3390/s24020492

**Published:** 2024-01-12

**Authors:** Yunze Shi, Peizhang Zhu, Tengyue Wang, Haonan Mai, Xiyang Yeh, Liangjing Yang, Jingfan Wang

**Affiliations:** 1 ZJU-UIUC Institute, International Campus, Zhejiang University, Haining 314400, China; yunze.20@intl.zju.edu.cn (Y.S.); tengyue.21@intl.zju.edu.cn (T.W.); haonan1.22@intl.zju.edu.cn (H.M.); 2 School of Mechanical Engineering, Zhejiang University, Hangzhou 310058, China; 3 Flexiv Ltd., Santa Clara, CA 95054, USA; peizhang.zhu@flexiv.com (P.Z.); xiyang.yeh@flexiv.com (X.Y.);; 4 Department of Mechanical Engineering, University of Illinois Urbana-Champaign, Urbana, IL 61801, USA

**Keywords:** virtual fixtures, 3D image-guided robots, virtual force field, human–robot collaboration

## Abstract

This paper proposes a method for generating dynamic virtual fixtures with real-time 3D image feedback to facilitate human–robot collaboration in medical robotics. Seamless shared control in a dynamic environment, like that of a surgical field, remains challenging despite extensive research on collaborative control and planning. To address this problem, our method dynamically creates virtual fixtures to guide the manipulation of a trocar-placing robot arm using the force field generated by point cloud data from an RGB-D camera. Additionally, the “view scope” concept selectively determines the region for computational points, thereby reducing computational load. In a phantom experiment for robot-assisted port incision in minimally invasive thoracic surgery, our method demonstrates substantially improved accuracy for port placement, reducing error and completion time by 50% ($p=1.06\times 10^{-2}$) and 35% ($p=3.23\times 10^{-2}$), respectively. These results suggest that our proposed approach is promising in improving surgical human–robot collaboration.

## 1. Introduction

The role of robots has expanded beyond task automation and autonomous planning to encompass increasingly collaborative roles in completing tasks alongside human operators and experts. This paradigm shift is particularly evident in the domain of medical robots; wherein, unlike typical industrial robots designed for manufacturing automation, the exclusion of human subjects is often not, if ever, an option. The human-centered nature of medical robot applications requires different sets of design specifications and safety considerations. Although control and planning techniques for human–robot collaboration are being developed by an increasing number of researchers [1,2,3], the interaction between humans, robots, and the environment and its feedback remains a challenge.

In recent years, there has been a large amount of research providing visual feedback for human–robot collaboration. However, providing visual feedback alone will have a significant impact on tasks that require a “hands-on” approach, such as in surgery, where the “feel in the hand” of the surgeon is very important to the success or failure of the surgery. Therefore, it is necessary to provide force feedback along with visual feedback.

### 1.1. Virtual Fixture

The term “virtual fixture” was initially coined by Rosenberg [4] as a shared control strategy. Just like using a ruler on paper to draw a straight line, virtual fixtures (VFs) can provide operators with effective haptic guidance and feedback. VFs are typically employed to guide users along task-specific paths or restrict user actions from unsafe areas. There have been numerous methods utilizing basic geometric shapes, such as points [5], lines, curves [6,7,8], planes [9], and geometric primitives [10,11], to generate VFs. However, in surgical tasks where the environment is dynamic and complex, it becomes tedious and impractical to manually select and define geometric primitives. The research on the dynamic virtual fixture is limited [12,13].

One approach is to utilize 3D anatomical structures to generate VFs. Indeed, the construction of anatomical structures for virtual fixtures often relies on preoperative information, such as medical imaging data [14]. The effectiveness of VFs is highly influenced by the level of detail and accuracy of the mesh used to represent the anatomical structures [15].

In [16], the implicit function of a surface was generated through a point cloud, and the virtual force field (VFF) was then used to create the interactive force. This method requires a calculated process to the implicit function of the surface, which poses a significant challenge to computational capacity and reduces timeliness [17]. A method that can better maintain the dynamic generation of virtual fixtures without the need for prior computations is to directly utilize point clouds. Yamamoto et al. [18] implemented a point cloud to generate VF, but only valid for static scenarios. In [19], a virtual fixture was proposed such that the tooltip verifiably never violates forbidden areas defined by the point cloud. However, the virtual fixtures are generated without considering the dynamic characteristics of the point cloud in these methods. In [17], a proxy method for the haptic rendering of streaming point cloud data was proposed. This is achieved without requiring any pre-processing of the points and without utilizing surface properties such as surface normals. However, one drawback of the proxy algorithm is that it may allow some degree of penetration through the surface of the forbidden region [12], which is unacceptable in surgical scenarios.

### 1.2. Port Incision in Robot-Assisted Minimally Invasive Thoracic Surgery

**Figure 1:**
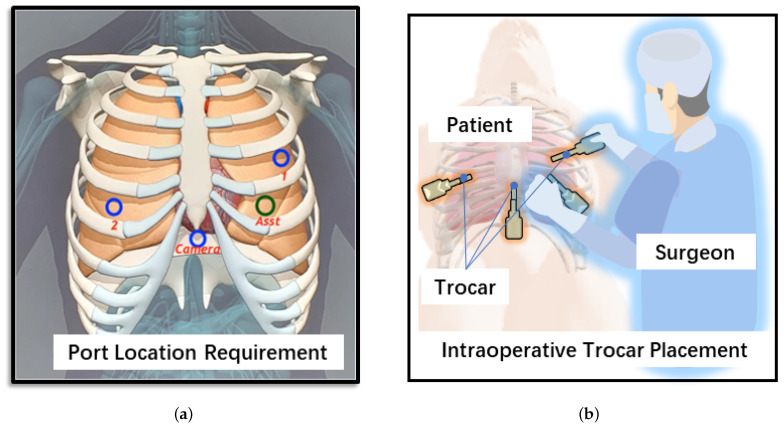
(**a**) Typical port location requirement [22]. (**b**) Intraoperative trocar port placement.

In modern surgical procedures, the practice of minimally invasive procedures is widely adopted to minimize damage to the body [20], as shown in Figure 1. Although robotic assistance is becoming popular in minimally invasive surgery, surgeons continue to perform the port incision operation independently by hand. During the drilling and puncturing procedure, surgical tools should be correctly aligned to the port while maintaining a safe distance from the human body; after drilling and puncturing, the trocar should be kept in the port position. Therefore, the use of robot-assisted incision not only provides accurate port placement but also frees up the surgeon’s hands for holding the trocar after drilling and puncturing. For example, in minimally invasive thoracic surgery, two of the more commonly used methods include two-port VATS (video-assisted thoracic surgery) and Uniportal VATS [21]. In the former, all surgical instruments use a shared port and the camera uses another tiny port. The latter involves sharing one port for the thoracoscope and surgical tools. These methods invariably require drilling and puncturing ports in the human body, which are generally located in the intercostal space determined using prior information.

Collaborative drilling and puncturing is a complex task as it requires the robot to provide both greater stiffness for drilling and puncturing and lower stiffness for aligning [23]. Moreover, switching between these stiffness levels needs to be performed intuitively, reliably, and rapidly, requiring proper detection and interpretation of the surgeon’s intention. This paper introduces a method based on VFF that does not require the identification of the surgeon’s intent. A novel approach is presented that leverages intra-operative depth information from an RGB-D camera to generate dynamic VF based on the VFF specifically aimed at assisting in the frequently encountered drilling and puncturing operation during minimally invasive surgery. The main contributions of this paper are listed as follows:

1. This method allows full utilization of the point clouds collected intra-operatively and does not require additional processing, such as creating implicit surface functions or estimating normal vectors;
2. In this paper, a novel force field function is proposed to generate a virtual fixture. Only a simple sigmoid function is used to implement both the forbidden virtual fixture (FVF) and guidance virtual fixture (GVF) generation by choosing different parameters. Due to the superposition of force fields, the algorithm does not require an explicit definition of transition regions or collision detection;
3. A “view scope” of contact points is established, and only the points within this scope exert guidance/forbidden forces on the operator, which not only excludes the influence of distant points but also reduces the computational burden of the algorithm;
4. This paper proposes a valuable framework and demonstrates the feasibility of using robot-assisted drilling and puncturing port incisions in minimally invasive thoracic surgery as a practical application. In minimally invasive surgeries, drilling and puncturing tasks often require identification of the surgeon’s intent [23]. However, the VF generation method proposed in this paper utilizes appropriate force fields, allowing the robot to guide the surgeon correctly without the need for intent recognition.

## 2. Proposed Framework

For the aforementioned surgical application for robot-assisted drilling and puncturing port incisions, the proposed system should possess some key features as shown in Table 1. Firstly, it should be capable of swiftly generating VFs without the need for prior information such as CT imaging data. Secondly, the VFs should be adept at adapting promptly to changes in the intra-operative environment, such as deformations caused by the patient’s respiration and heartbeat. Thirdly, the system should be able to precisely generate shapes that conform to the contours of the patient’s body, thereby preventing undesired collisions and injuries. Lastly, the system should seamlessly transit between behaviors such as aligning, drilling and puncturing without necessitating explicit identification of the surgeon’s intent.

**Figure 2:**
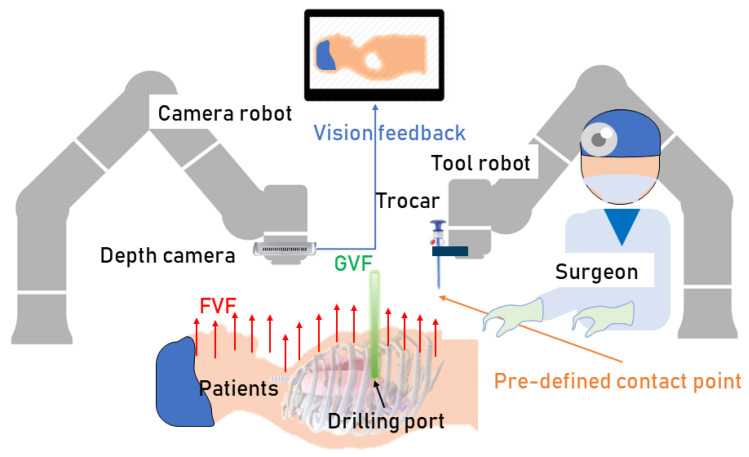
The system of robot-assisted drilling and puncturing.

**Figure 3:**
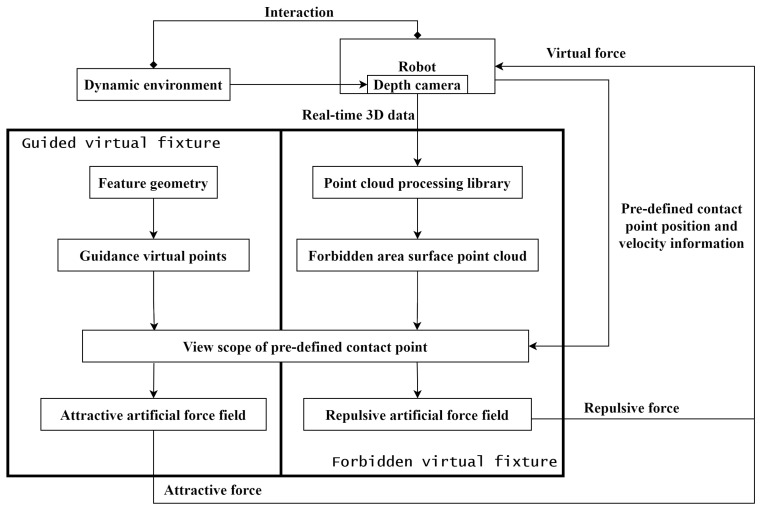
Framework of the proposed dynamic virtual fixture generation. The camera collects dynamic environmental information in intra-operation and forms point cloud data. Through the algorithm proposed in this paper, the intra-operative point cloud generates a virtual fixture, which generates an attractive or repulsive force to guide the interaction of the operator with the environment.

As shown in Figure 2, this study proposes a generalized framework to dynamically generate a virtual fixture using intra-operative 3D image feedback from a depth camera. The 3D images of target objects in this study are captured by an RGB-D camera and handled by the 3D data-processing library Open3D to generate a point cloud for use in virtual fixture generation. Herein, the target object is the deformable soft tissue in surgical applications. A spherical VFF is generated around each point in the online generated point cloud which is in the view scope. Based on the distribution and field parameters of VFFs, FVFs and GVFs can be configured according to the intended application. Eliminating the need for intent recognition, automatic transitioning between aligning, drilling and puncturing behaviors can be achieved through the differential force field effects generated by FVF and GVF. We propose a novel concept called the pre-defined contact point (PDCP). This concept is different from that of the Tool Center Point (TCP) in robotics terminology. TCP refers to the center point of the actual tool installed on the robot. In contrast, the proposed PDCP in this study is a virtual point that can be adjusted based on different operations, without the necessity of corresponding to a real tool. If there are offsets or errors in the point cloud acquired by the camera, we can compensate for them by adjusting the position of the PDCP. In addition, for safety reasons during surgery, the PDCP may also be defined at a virtual position on a presumably mounted surgical tool. Additionally, the PDCP has a defined scope of view. Only the points within this field of view contribute to the generation of the VF. The framework of the system is shown in Figure 3.

## 3. Dynamic Virtual Fixture Generation

### 3.1. Intra-Operative Point Cloud Acquisition

During runtime, the depth camera continuously captures new 3D images, which are then processed by a library such as Open3D to generate a point cloud that accurately represents the current state of the target object, in this case, the surface of the human body. This capturing–processing–generation process is set to run periodically at a specific frequency, which is determined by factors such as camera frame rate and computational capacity. The proposed method is not highly dependent on the density of the collected point cloud, as long as the radius of the spherical force field is larger than the distance between two adjacent points to ensure that the force field is continuously close to the surface shape of the object. However, point cloud density has to be appropriately specified to ensure efficient and accurate performance.

### 3.2. VF Generation Methods

#### 3.2.1. FVF Generation

**Figure 4:**
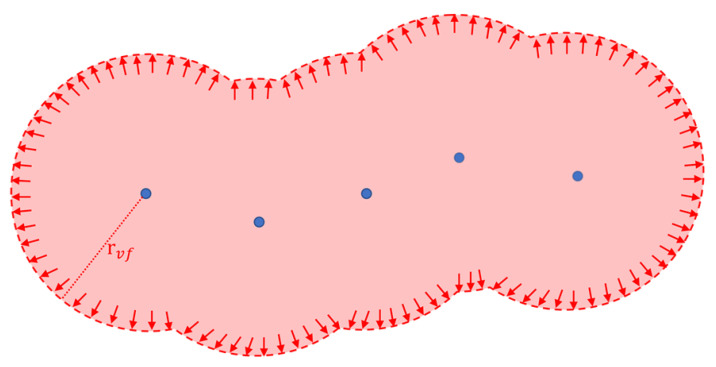
2D illustration of the forbidden-region virtual fixture based on the superimposed virtual force field. The blue points represent the points in the point cloud. Each point in the point cloud forms a spherical virtual force field, and a continuous force field is generated under the superposition of adjacent force fields. The repulsive force is concentrated in the inner part of the forbidden region as the red arrows show, repelling any movement into the interior.

The FVF is designed to prevent the PDCP from penetrating the surface of an object. To achieve this, for each point in the point cloud of the object, a spherical force field is added such that the point is the center of the field. Each point can be represented as a repulsive force source. The force fields from the points in the view scope are fused into a continuous wall by force superposition. Furthermore, when the PDCP approaches any part of this wall, the wall generates a repulsive force to prevent the contact point from penetrating, as shown in Figure 4. $r_{fvf}$ represents the radius of the spherical force field, which is decided by the parameters of the VFF function.

**Figure 5:**
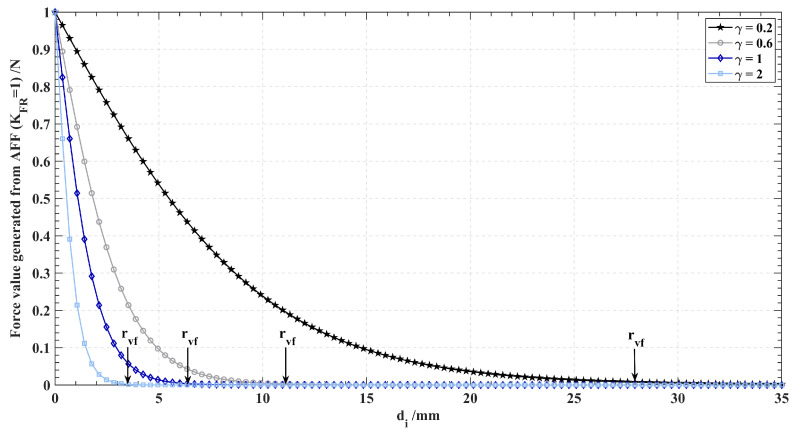
Characteristics of the sigmoid function with different γ (for only the positive half-axis). For $d_{i}$ greater than $r_{vf}$, the force generated by the force field approaches zero.

The spherical repulsive force field generation is based on a modified one-sided sigmoid function as represented in (1), which is related to $d_{i}\left(/mm\right)$, the distance from PDCP to the *i*th point $p_{i}$ in the point cloud.

(1) $$\begin{array}{ccc} \vec{F_{rep,i}\left(d_{i}\right)} & = & \frac{2K_{FR}}{1+e^{\gamma_{rep}\cdot d_{i}}}\cdot \frac{\vec{X_{PDCP}}-\vec{X_{p_{i}}}}{d_{i}}, \end{array}$$

where $F_{rep,i}$ is the repulsive force generated by the $i_{th}$ point. $\gamma_{rep}$ is a parameter related to the $r_{vf}$ that controls the influence range of VFF. The characteristics of the $F_{rep,i}$ model for different $\gamma_{rep}$ values are shown in Figure 5. As $\gamma_{rep}$ increases, $r_{fvf}$ becomes smaller and the force change becomes steeper. When $d_{i}$ is approaching zero, the magnitude of the force is the largest ($K_{FR}$). For different application requirements and different robot stiffnesses, different $K_{FR}$ values can be selected to control the maximum force. $\vec{X_{PDCP}}$ is the position of PDCP. $\vec{X_{p_{i}}}$ is the position of $p_{i}$. The force is considered to be absent when it is less than a very small value ϵ, which depends on the stiffness of the chosen arm. From this, $r_{fvf}$ can be calculated as:

(2) $$\begin{array}{ccc} \frac{2K_{FR}}{1+e^{\gamma_{rep}\cdot r_{fvf}}} & = & \epsilon , \end{array}$$

(3) $$\begin{array}{ccc} r_{fvf} & = & \frac{\ln[\left(2K_{FR}-\epsilon \right)/\epsilon ]}{\gamma_{rep}}. \end{array}$$

Assume that the maximum distance between every two neighboring points in the point cloud is $d_{p}$. To ensure that the force field fits the object surface shape better and to ensure the continuity of the force field, $\gamma_{rep}$ must be adjusted to ensure that $r_{fvf}$ is greater than $d_{p}$.

(4) $$\begin{array}{c} r_{fvf}=\frac{\ln\frac{2K_{FR}-\epsilon }{\epsilon }}{\gamma_{rep}}>d_{p}, \end{array}$$

(5) $$\begin{array}{c} \gamma_{rep}<\frac{\ln[\left(2K_{FR}-\epsilon \right)/\epsilon ]}{d_{p}}. \end{array}$$

The total repulsive force $\vec{F_{rep}}$ represents the superposition of the forces generated by *n* points in the current view scope as (6).

(6) $$\begin{array}{ccc} \vec{F_{rep}} & = & \sum_{i=0}^{n}\vec{F_{rep,i}\left(d_{i}\right)}. \end{array}$$

This method of generating a VFF based on a local point cloud ensures the dynamic generation of a virtual fixture. When the object is moving, rotating, or deforming, the collected point cloud also changes such that each spherical force field also changes. Consequently, the generated FVF can keep pace with the changes of the target object.

#### 3.2.2. GVF Generation

Compared with an FVF, a GVF is mainly used for guidance rather than for constraint operation. This study showcases the attraction of a PDCP onto a GVF using an attractive force field such that the manual operation of a human operator is guided by the virtual force.

Point cloud segmentation technology uses the captured point cloud to identify geometrical features, such as circles, planes, cylinders, wedges, and squares. The GVF can be established based on the geometric characteristics of identified geometries and practical applications. Although the definition of the GVF shape is not within the scope of this study, the method for generating the guiding force is an important consideration. Assuming that open-source software is used to identify the feature geometry, the useful geometric features are determined in advance according to the intended practical application. In this work, the task involves a drilling and puncturing operation, and therefore, a tubular GVF is assumed to be formed using a straight line as the geometric feature. The goal is to achieve unconstrained motion along the axial direction inside the tube while limiting, to some extent, the radial motion trying to move out of the tubular GVF. The GVF is generated by performing a force-field superposition like the FVF. A series of virtual points spaced at $d_{vp}$ are added to the line, and each point generates a spherical force field that is superimposed to form a tubular region.

To solve the aforementioned problem, the selected attractive field should have the following characteristics [24]: firstly, it should avoid exerting a large force when the tool is far from the desired GVF axis. This ensures that the interaction forces are proportional to the proximity to the desired axis, providing more intuitive and gentle guidance. Secondly, it should avoid applying excessive force when the tool is close to the GVF axis. This prevents the generation of overly restrictive or strong oscillations, allowing for more flexibility and adaptability in surgical tasks. This is precisely what collaborative drilling and puncturing operations require: higher stiffness for drilling and puncturing and lower stiffness for alignment. To satisfy these features, a near-large and far-small sigma field with a small force-free shell can be used, as represented by (7):

(7) $$\begin{array}{c} \vec{F_{att,i}\left(d_{i}\right)}=\left\{\begin{array}{c} \frac{2K_{FA}}{1+e^{\gamma_{att}\cdot d_{i}}}\cdot \frac{\vec{X_{p_{i}}}-\vec{X_{PDCP}}}{d_{i}}\;d_{i}>r_{s}\\ 0\;\;\;0\leq d_{i}\leq r_{s}, \end{array}\right. \end{array}$$

where $F_{att,i}$ is the attractive force generated by the VFF at the $i_{th}$ point in the PDCP view scope. γ has the same properties as in (1), affecting the range of influence $r_{gvf}$ of the attractive field. $K_{FA}$ is the maximum value of the force generated by a single attractive point. $r_{s}$ is the radius of the small shell around the attractive point. Note that the parameter $r_{s}$ needs to be appropriately set to achieve two objectives. Firstly, it ensures that there are no noticeable abrupt changes in force after superposition. This implies that the transition from zero force to applied force should be smooth and seamless, ensuring a continuous and consistent force experience for the user. Secondly, the shell radius should be chosen such that it encompasses adjacent points, allowing for a continuous force vacuum region. This ensures that the virtual fixture can effectively guide the user while maintaining a consistent and uninterrupted force field. The methodology for determining the appropriate parameter values will be discussed in detail in the subsequent sections.

The required GVF can be obtained by vector superposition of the force fields. Because of the implementation of the empty shell, the monotonicity of the force after superposition meets the requirements of the GVF. The nearest one or two attractive points to the PDCP are defined as $P_{close}$ and the other points as $P_{others}$, which generate the forces $\sum \vec{F_{att,close}}$ and $\sum \vec{F_{att,others,i}}$, respectively. Because the points are sufficiently dense, the radial distance between the PCDP and GVF can be considered as its distance $d_{close}$ from $P_{close}$, and the distance between $P_{close}$ and the *i*-th point in $P_{others}$ is denoted as $d_{co,i}$. The attractive force can be expressed as:

(8) $$\begin{array}{ccc} \vec{F_{att}} & = & \vec{F_{att,close}}+\sum \vec{F_{att,others,i}}. \end{array}$$

**Figure 6:**
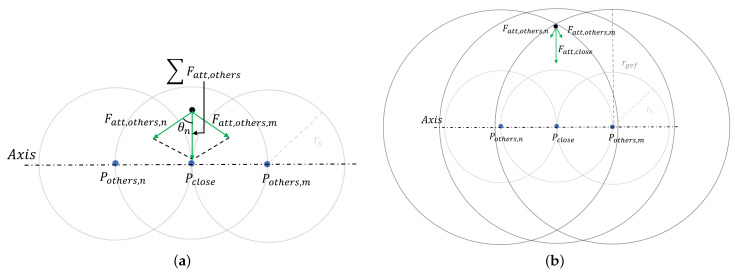
Two cases for the change in the radial attractive force. The attractive points are considered more intensively than those in the schematic, but the effect of force superposition is the same. (**a**) Case 1: PDCP is inside the GVF. (**b**) Case 2: PDCP is outside the GVF.

**Figure 7:**
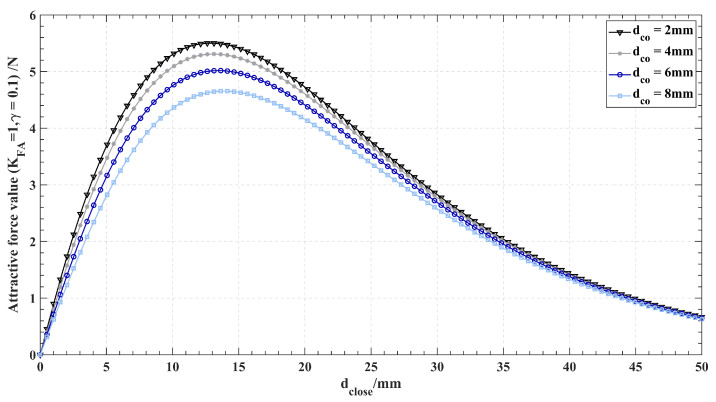
Relationship between $F_{att,others,i}$ and $d_{close}$. As the distance increases, the force increases till the peak point and decreases after.

Since the points on the line are aligned along the axial direction, the axial component of the superimposed force is approximately zero due to the symmetry of the points, which achieves free axial motion inside the GVF. Therefore, the attractive force is mainly concentrated in the radial direction, and two cases exist for the change in the radial attractive force after superposition. In the first case, when the PDCP enters the force vacuum of $P_{close}$, $F_{att,close}$ is 0 N, and some $P_{others}$ always exist such that the PDCP does not enter their vacuum, as shown in Figure 6a. When the PDCP is considered to have entered the GVF, the attractive force is expressed as (9):

(9) $$\begin{array}{cc} \vec{F_{att}} & =\sum \vec{F_{att,others,i}}\\ \; & =\sum ∥\vec{F_{att,others,i}}∥\cos\theta_{i}\vec{n_{i}}\\ \; & =\sum \frac{2K_{FA}}{1+e^{\gamma_{att}\cdot \sqrt{d_{close}^{2}+d_{co,i}^{2}}}}\frac{d_{close}}{\sqrt{d_{close}^{2}+d_{co,i}^{2}}}\vec{n_{i}}, \end{array}$$

where $\vec{n_{i}}$ represents the direction vector of each force after superposition. $\theta_{i}$ is the angle between $F_{att,others,i}$ and the radial direction. After the parameters ($\gamma_{att}$ and $K_{FA}$) of the force field are determined for every single point, the relationship between $F_{att,others,i}$ and $d_{close}$ is shown in Figure 7. Note that the trend of the force generated by each point in $P_{others}$ is the same in the case of a dense distribution of attractive points. As the distance between the PDCP and axis increases, the attractive force generated by $P_{others}$ increases till the peak point and decreases after. By setting the radius $r_{s}$ of the force vacuum zone to a value corresponding to the distance from the peak point, the monotonic increase of the attractive force within the vacuum zone of $P_{close}$ is ensured without affecting the monotonically decreasing trend of $F_{att,close}$ outside the vacuum zone.

In the second case, the PDCP does not enter the vacuum area of any point, as shown in Figure 6b. In this case, the attractive force originates from the forces generated by $P_{close}$ and $P_{others}$, and this force increases as the distance decreases, thus guiding the operator into the force vacuum of $P_{close}$.

The aforementioned discussion showcases that the design goals and requirements of the GVF can be achieved. To elaborate, the attractive force is near-large and far-small outside the GVF, and the PDCP is guided into the interior of the GVF when it is close to the GVF. The attractive force is close to 0 N when it is close to the axis and the attractive force is larger when it is far from the axis. Moreover, the attractive force exists only in the radial direction, and free motion can be achieved in the axial direction.

#### 3.2.3. View Scope of PDCP

**Figure 8:**
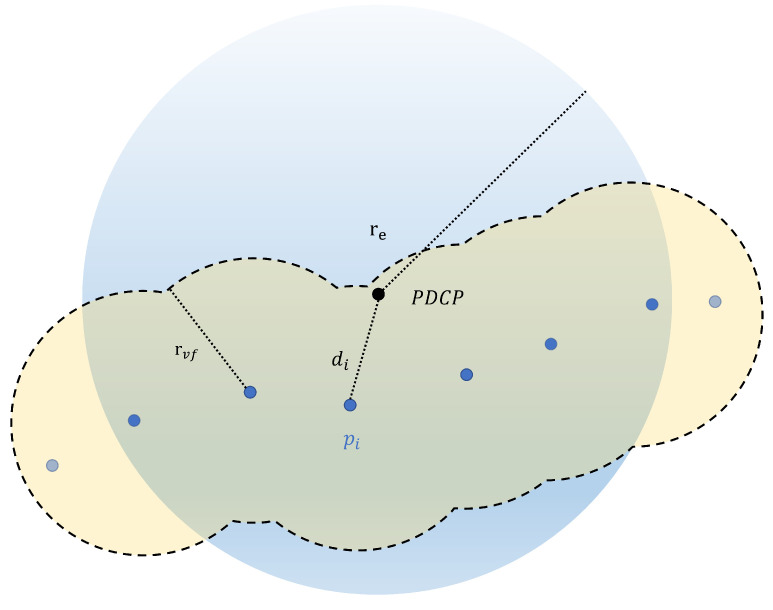
Two-dimensional illustration of the view scope of PDCP. Only the points in the view scope generate force fields.

To avoid the problem of high computational costs caused by excessive point cloud data and the influence of the field generated from distant points on the current position, the VFF is generated only at the points set near the PDCP because only these points are effective for the generation of the virtual fixture, and other points can be ignored. In addition, if the point cloud density is too low, it can be interpolated within this scope. As shown in Figure 8, the proposed method does not require the application of various search algorithms but creates a virtual spherical cavity centered on the PDCP to represent the view scope, and only the point $p_{i}$ satisfying $d_{i}\leq r_{e}$ will generate a force field, where $r_{e}$ denotes the radius of the cavity around the PDCP, which is related to the velocity of the PDCP as:

(10) $$\begin{array}{ccc} r_{e} & = & ∥v_{e}∥\cdot k_{v}+r_{0}, \end{array}$$

where $v_{e}$ is the velocity of the PDCP. $k_{v}$ is an appropriate scale factor. A high velocity indicates that fast and large-scale operations need to be performed. Therefore, the cavity radius also increases accordingly, leading to more points to generate force fields, ensuring greater coverage of the virtual fixture. On the contrary, when the velocity is low, some fine micro-operations need to be carried out. In this case, the cavity radius is small, and the virtual fixture is more concentrated in a specific area. $r_{0}$ is the basic radius of the cavity. In order to avoid sudden changes in force, it must be ensured that $r_{vf}<r_{0}$.

(11) $$\begin{array}{c} \frac{\ln[\left(2K_{F}-\epsilon \right)/\epsilon ]}{\gamma }<r_{0}, \end{array}$$

(12) $$\begin{array}{c} \gamma >\frac{\ln[\left(2K_{F}-\epsilon \right)/\epsilon ]}{r_{0}}. \end{array}$$

Based on (5), the γ for generating the FVF/GVF should be set to satisfy:

(13) $$\begin{array}{c} \frac{\ln[\left(2K_{F}-\epsilon \right)/\epsilon ]}{r_{0}}<\gamma <\frac{\ln[\left(2K_{F}-\epsilon \right)/\epsilon ]}{d_{p}}, \end{array}$$

where the $d_{p}$ is the maximum distance between every two neighboring points in the point cloud for the FVF and the distance between attractive points for the GVF.

The parameter γ in the VF generation function needs to be determined by a process of value-tuning in the value range. The median value of the upper and lower bounds of the range can be taken first and then fine-tuned. This γ value can be readily tuned based on the experiment setting and readjustment of the new value is not required as long as the configuration of the camera and VF used remains unchanged.

## 4. Results and Discussion

In this section, the performance and wide applicability of the proposed dynamic VF generation algorithm are demonstrated for practical applications in surgical procedures.

### 4.1. Experimental Setup

**Figure 9:**
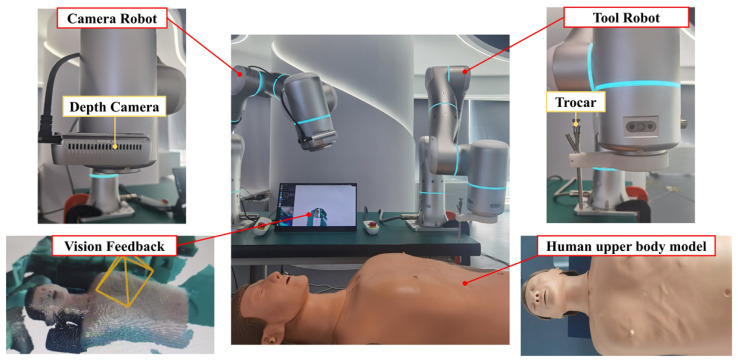
The actual setup of robot-assisted drilling and puncturing experiments.

As shown in Figure 9, in this experiment, the practicality of the proposed dynamic virtual fixture was demonstrated on a mock-up patient model simulating the procedure of thoracic port incision. A system of two Flexiv Rizon 4 robot manipulators with translational Cartesian stiffness of 4000 N·m and rotational Cartesian stiffness of 250 N·m/rad were used, namely a camera robot and a tool robot. The camera robot was mounted with an Intel RealSense D435 depth camera and was dedicated to intra-operative depth image capture for generating the point cloud. The tool robot was dedicated to operations using a trocar tool. As the depth camera has a fixed field of view and an optimal range of distance as well as to guarantee consistent quality of the captured depth images, the depth camera was mounted on a separate robot instead of on the same robot with the tool such that the movement of the tool robot will not affect the intra-operative image-capturing process. For a typical setup, the camera and tool robots were mounted side-by-side with a known base offset, which can vary to fit the rest of the surgical setup. The point cloud generated and processed by the camera robot was transformed into the coordinates of the tool-manipulating robot, which then used the transformed point cloud to generate virtual fixtures.

In the actual surgical workflow, the trocar is not pre-installed on the robot to ensure safety. The robot needs to be moved to the target position first, followed by the assembly of the trocar. In this configuration, to facilitate the process of guiding the robot to a position where the trocar can be directly installed, the PDCP can be defined at the tip position of the assumed mounted trocar. It is important to note that in the subsequent experiments of this study, the trocar is directly mounted on the robot. This decision is made to demonstrate the avoidance of risk under a consistent and controlled experimental environment.

The FVF was generated from the intra-operative point cloud to produce a repulsive force when the PDCP is close to the human body surface to avoid the tooltip from injuring the human body. For the GVF, a series of attractive points was added above the port location to produce a tubular guidance area that created an attractive force when the PDCP approached the top of the port, allowing the tool to move freely up and down within the tube to facilitate puncture. In contrast, the GVF created an attractive force to guide the operator back into the tube whenever the operator attempts to leave the tube. The 3D image acquisition and processing ran at 100 FPS, and the robot control and virtual fixture generation ran at 1 kHz. If the point cloud information was not updated, the VF generation would follow the data of the previous graphics frame. The parameters used in the experiment are listed in Table 2.

### 4.2. FVF Evaluation

The FVF was generated using the point cloud of the human surface collected intra-operatively from the depth camera mounted on the camera robot. Repulsive forces were generated by the FVF when the PDCP on the trocar was close to the human body. To simulate the deformation of the human surface due to breathing and heartbeat and to demonstrate that the virtual fixture is dynamic, a Gaussian float with a period of 3 s was added to the human surface.

**Figure 10:**
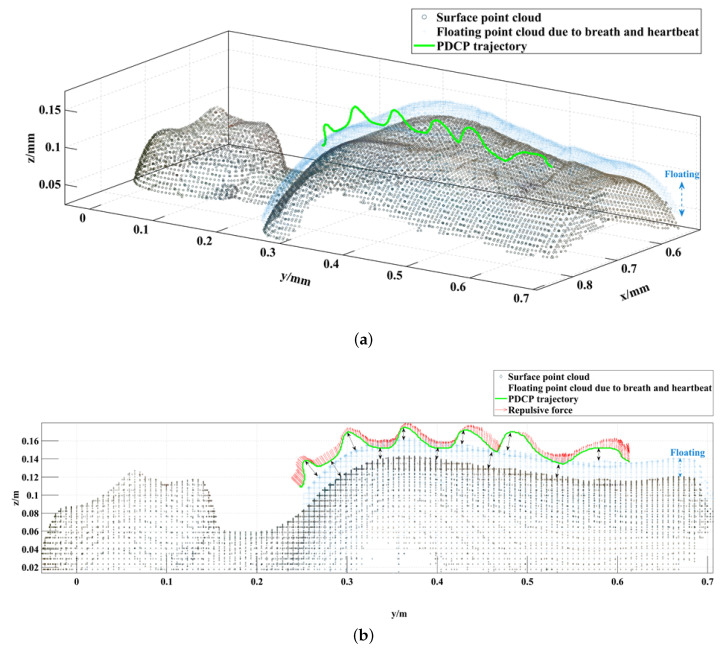
Experimental effect of the dynamic FVF. (**a**) Motion trajectory of PDCP in 3D view. (**b**) PDCP trajectory in the Y–Z plane and the repulsive force on the tool robot.

In the experiment, the tool robot was grasped, moved along the manikin surface from the abdomen to the neck, and continued trying to move downwards. Certainly, real-world robotic surgical scenarios do not involve such risky operational procedures. However, the primary objective of this experiment is to qualitatively validate the effectiveness of the proposed method. It aims to demonstrate that even in extreme and potentially risky situations, the introduced FVF can ensure patient safety. The effects of the dynamic FVF can be demonstrated by the exerted forces from the FVF and PCDP position. As shown in Figure 10, under the effect of the FVF, a repulsive force of 35.95 ± 14.51 N ([21.44 N, 41.76 N]) was practically exerted on the tool robot. The PDCP located on the tip of the trocar maintained a distance of 41.0 ± 5.3 mm ([35.7 mm, 44.3 mm]) from the moving body surface. By generating a dynamic FVF, the surgical tool was kept at a safe distance of at least 35 mm from the body while avoiding the need to impose a large exclusion zone that would restrict the surgeon’s operating area. Furthermore, the FVF guarantees a risk-free operation even with the trocar installed. This underscores the potential of our approach to streamline the current two-step surgical workflow into one, demonstrating its capabilities to be generalized for rapid and precise robot-assisted trocar placement beyond thoracic surgical procedures.

### 4.3. GVF Evaluation

The GVF was a cylindrical tube built at the drilling and puncturing port, with the port as the center point of its cross-section. A tubular guiding region can be generated by adding a series of attractive points along the axis of the cylinder and applying the aforementioned method to generate a GVF. Port location can be determined by preoperative inspection and intraoperative alignment, which is outside the scope of this study. Therefore, the drilling and puncturing port in this experiment was selected based on our hypothesis for the human skin surface.

**Figure 11:**
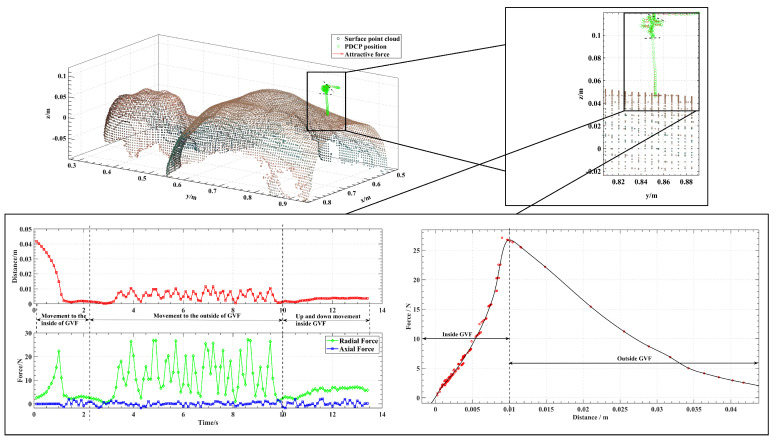
Experimental effect of the GVF in which distance refers to the distance between the current PDCP position and the axis of the tube.

As shown in Figure 11, the attractive force was concentrated in the radial direction, whereas the axial direction was not constrained, which allows for free up-and-down motion inside the GVF for port incisions. When the PDCP is outside the GVF, no force is generated if the PDCP is far from the GVF axis, while when closer, the attractive force leads the operator to the inside of the GVF. When the PDCP is within the GVF, the attractive force will act to guide the operator back towards the axis if they attempt to move outside of the GVF. Similarly, when moving vertically (up and down), the attractive force will guide the operator to maintain the PDCP on the axis, ultimately allowing the PDCP to reach the designated port. From the distance–force relationship, note that the actual GVF generated is consistent with the design, and as the distance between the tool and axis increases, the force gradually increases inside the GVF and decays outside the GVF. This demonstrates that the generated GVF in this paper can achieve higher stiffness for drilling and puncturing and lower stiffness for aligning without the need to identify human intent.

### 4.4. Results of VF-Assisted Drilling and Puncturing Port Incision Tasks

**Figure 12:**
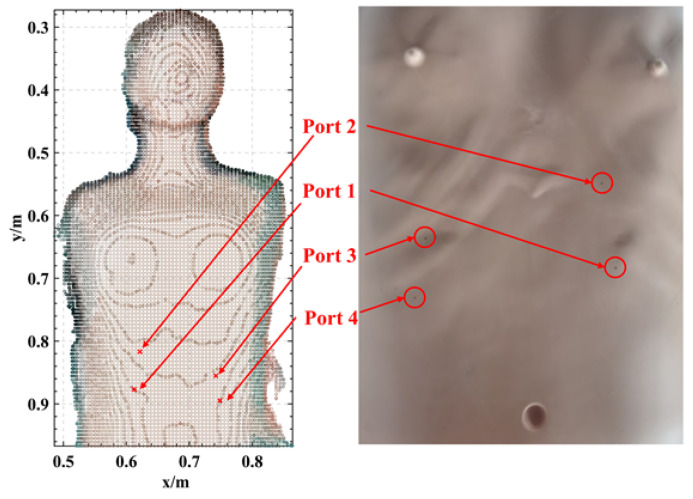
Four points are selected on the human chest as drilling and puncturing ports to simulate minimally invasive thoracic surgery.

In the experiments described in this subsection, six volunteers were selected as experimental subjects to perform a simulated port incision operation. The ports were selected four on the human chest and each port was labeled, as shown in Figure 12. The volunteers started from the same starting point and operated the tool robot with/without the assistance of the virtual fixture to move to the markers of each port while avoiding other parts of the human body, and this process was considered to encompass one experiment. The experiment was repeated five times by each volunteer independently. Port incision error and completion time are considered performance parameters in the evaluation of the system. The completion time is the average time taken to complete the positioning of each port. Each port has a known exact position before the experiment (in the robot’s base coordinate system), and during the experiment when the subject thinks that he/she has reached the port position he/she will step on the pedal switch with their foot, and at this time the robot will record the position of the end of the tool (the robot arm used for the experiment has a localization error of 0.1 mm), and from this, the port incision error will be calculated.

**Figure 13:**
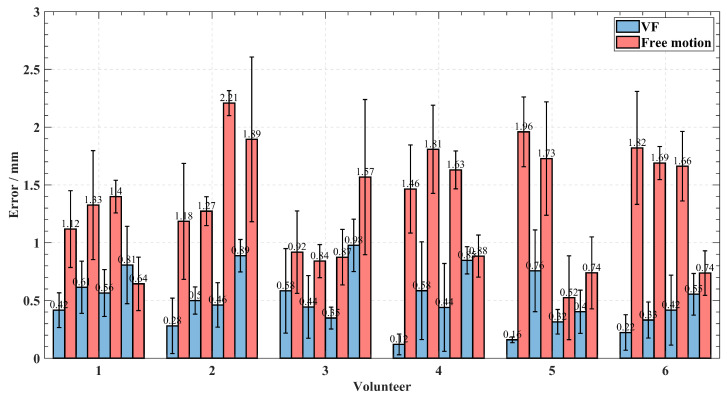
Mean errors of each volunteer drilling or puncturing each port with standard error bars.

Figure 13 shows the mean values of the port incision errors from each port during the free motion and virtual fixture assistance tasks. Error bars represent the standard errors of the means. As evident from Table 3, with the assistance of a virtual fixture, the average port incision error is reduced from 1.33 mm in free motion to 0.50 mm, and the average completion time is reduced from 13.93 s in free motion to 7.93 s. To demonstrate the statistical validity of the results, port incision errors with/without virtual fixture assistance are compared using a paired t-test at a significance level of α=0.05. The t-test results suggest that the port incision error is reduced 50% ($p=1.06\times 10^{-2}$). Completion time is reduced by 35% ($p=3.23\times 10^{-2}$) with the aid of the virtual fixture compared to free motion.

**Figure 14:**
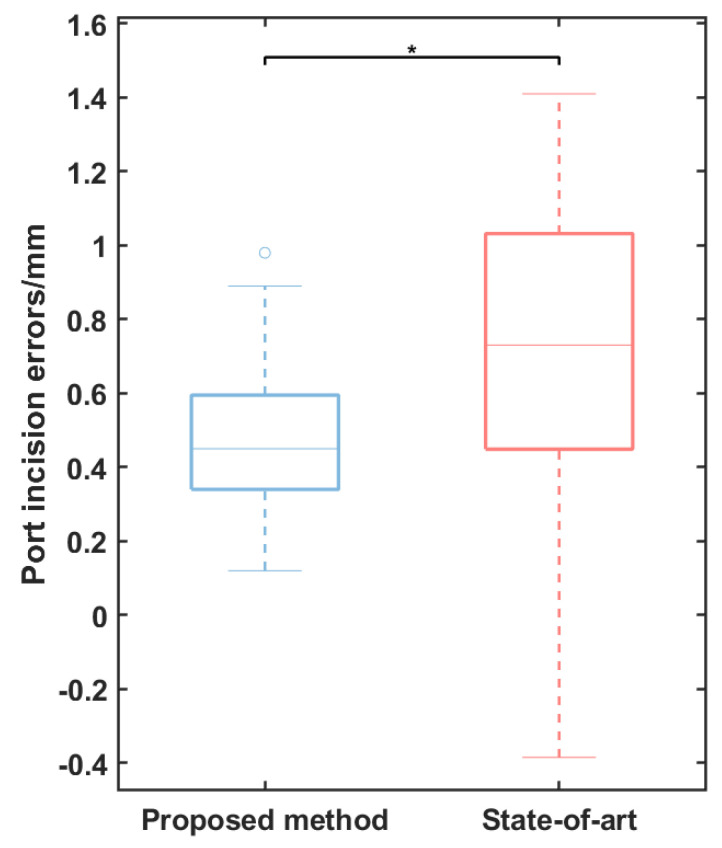
A comparison of the incision errors between our proposed method and the state-of-the-art method utilizing geometric primitives based on human intent recognition. (*, *p* < 0.05).

The observed average error in port incision is 0.50 mm with a standard deviation of 0.20 mm. In comparison, the state-of-the-art method utilizing geometric primitives based on human intent recognition [23] reports an average incision error of 0.78 mm with a standard deviation of 0.50 mm. As illustrated in Figure 14, there exists a statistically significant difference (*p* < 0.05) between our proposed approach and the state-of-the-art method based on human intent recognition. This indicates that the methodology proposed in our study is capable of achieving more accurate and controlled port incisions. This improvement is particularly crucial in surgical contexts, where precision is paramount. The proposed method, by mitigating errors, not only enhances the safety of the procedure but also contributes to the overall efficiency of robotic-assisted surgeries. Furthermore, the ability of the proposed method to achieve lower error rates without reliance on human intent recognition underscores its robustness and autonomy. This is a critical advantage, as it reduces the complexity of the system and eliminates potential sources of error associated with human intent interpretation.

From the experimental results, it can be seen that the method proposed in this paper provides good assistance for the punching and positioning steps in minimally invasive thoracic surgery. The FVF can avoid injury to the patient’s body caused by surgical tools, and the GVF can provide guidance for the positioning of the surgeon, improve punching accuracy, and reduce operation time, which can reduce the burden placed on the surgeon during surgery.

## 5. Conclusions and Future Works

This study proposes a novel generalized framework for dynamic virtual fixtures generated from a point cloud utilizing the VFF method. The applications of this framework extend across various domains, including modern surgery and scenarios that demand close collaboration between humans and robots. In this study, 3D images acquired intra-operatively are used as feedback, and a modified VFF method is used to generate a dynamic FVF, which complies with the surface geometry of the object without the need for any prior knowledge or model, followed by the incorporation of a GVF to achieve guidance and obstacle avoidance. The proposed method is evaluated by conducting experiments based on a phantom model, demonstrating the effectiveness and practicality of the proposed method. Additionally, in the realm of human–robot collaboration in medical robotics, the proposed method facilitates the adjustment of VF stiffness without requiring identification of the surgeon’s intentions. This empowers surgeons to maintain autonomy during operations, fostering enhanced precision and reduced workload through collaborative efforts with the robot.

Despite the successful demonstration of the functionality and effectiveness, there exist limitations to be addressed. Firstly, the parameters in the force field function need to be determined over a small range of values, hence requiring a brief adjustment procedure before the operation. We will try to add the function of self-tuning in subsequent work. Secondly, the method proposed in this paper mainly uses the force formed by the superposition of the force field to guide the operator and does not analyze the effect of the moment formed by this force on the direction of the tool, while in fact the operator will control the direction of the tool, so the effect is not significant. We will also analyze and discuss this issue in future work. Thirdly, since the focus of this work is on the dynamic generation of VFs, the dynamic effects are manually added to the point cloud in the experiments, and in practice, the real-time collected point cloud will be inevitably obscured by the tool. Our future work will investigate better occlusion handling to realize the complete acquisition of the dynamic point cloud. We envision the extension of our development for the teleoperation of surgical procedures with improved human–robot collaboration in terms of interactivity and intuitiveness.

## Figures and Tables

**Table 1:** Comparison of various common methods for VF generation.

	Dependency on Pre-Operative Information	Convenience of Dynamic Updates during Surgery	Consistency with Irregular Body Surfaces in Surgery	Ease of VF Stiffness Transformation
Geometric primitives	High	Inconvenience	Low	Difficult
Anatomical structures	High	Inconvenience	High	Human Intent Recognition Required, Moderately Challenging
Implicit functions	High	Good	High	Human Intent Recognition Required, Moderately Challenging
Point clouds	Low	Convenience	Good	Virtual Force Fields Can Be Utilized, Relatively Easy

**Table 2:** Parameters used in the experiment.

Symbol	Parameter	Value
$K_{FR}$	Maximum value of the force generated by a single spherical repulsive force field	40 N
$K_{FA}$	Maximum value of the force generated by a single spherical attractive force field	8 N
$\gamma_{rep}$	Parameter in sigmoid function for repulsive force field	0.25
$\gamma_{att}$	Parameter in sigmoid function for attractive force field	0.17
$r_{s}$	Radius of the small shell around an attractive point	0.9 mm
$r_{0}$	Basic radius of the cavity	50 mm
$k_{v}$	Scale factor describing the relationship between cavity radius and PDCP velocity	0.5

**Table 3:** Port incision errors and completion time.

Volunteer		Port 1 (mm)	Port 2 (mm)	Port 3 (mm)	Port 4 (mm)	Mean (mm)	Completion Time (s)
VF assistance	Volunteer 1	0.42	0.61	0.56	0.81	0.60	8.42
	Volunteer 2	0.28	0.50	0.46	0.89	0.53	7.51
	Volunteer 3	0.58	0.44	0.35	0.98	0.59	8.10
	Volunteer 4	0.12	0.58	0.44	0.85	0.50	8.03
	Volunteer 5	0.16	0.76	0.32	0.40	0.41	7.58
	Volunteer 6	0.22	0.33	0.42	0.55	0.38	7.92
	Mean (mm or s)	0.30	0.54	0.43	0.75	0.50	7.93
Free motion	Volunteer 1	1.12	1.33	1.40	0.64	1.12	15.25
	Volunteer 2	1.18	1.27	2.21	1.89	1.64	15.08
	Volunteer 3	0.92	0.84	0.87	1.57	1.05	12.57
	Volunteer 4	1.46	1.81	1.63	0.88	1.45	10.81
	Volunteer 5	1.96	1.73	0.52	0.74	1.24	13.73
	Volunteer 6	1.82	1.69	1.66	0.74	1.48	16.16
	Mean (mm or s)	1.41	1.45	1.38	1.08	1.33	13.93

## Data Availability

Data are contained within the article.

## Abbreviations

The following abbreviations are used in this manuscript:

VF	Virtual fixture
VFF	Virtual force field
FVF	Forbidden virtual fixture
GVF	Guidance virtual fixture
VATS	Video-assisted thoracic surgery
TCP	Tool center point
PDCP	Pre-defined contact point
